# Pericarditis of Polyarteritis Nodosa

**DOI:** 10.7759/cureus.46717

**Published:** 2023-10-09

**Authors:** Robert Ambrogetti, Omer Taha, Baseer Awan, Ahsan Memon, Kehinde O Sunmboye

**Affiliations:** 1 Medicine, University Hospitals of Leicester NHS Trust, Leicester, GBR; 2 Rheumatology, University Hospitals of Leicester NHS Trust, Leicester, GBR; 3 Health Sciences, University of Leicester, Leicester, GBR

**Keywords:** rheumatology, cardiology, chest pain syndrome, polyarteritis, pericarditis

## Abstract

Polyarteritis nodosa (PAN) is a rare systemic vasculitis characterised by necrotising inflammation of medium-sized arteries. PAN can affect patients of any age, gender, or ethnic background. Its highest incidence is in the fifth-sixth decade of life, with a slight male-to-female predilection. PAN can be idiopathic or secondary to a multitude of systemic conditions, such as infection, haematological malignancy, or autoinflammatory disorders. PAN has a broad spectrum of possible clinical manifestations the most common being constitutional symptoms, such as fever and myalgia. While cardiac involvement is well-described and is a common cause of mortality, it is exceedingly uncommon as the initial presentation. Below, we describe a case of a female in her 60s who presented with pericarditis as the first manifestation of PAN.

## Introduction

Polyarteritis nodosa (PAN) is a rare systemic vasculitis characterised by necrotising inflammation of medium-sized arteries. Small branching arteries can sometimes be involved. However, arterioles, venules, and capillaries are spared. PAN can affect patients of any age, gender, or ethnic background. Its highest incidence is in the fifth-sixth decade of life with a slight male-to-female (1.5:1) predilection. PAN has a prevalence of around 33 per million and an incidence of 0-1.6 cases per million per year [1,2].

PAN can be idiopathic or secondary to a multitude of systemic conditions, such as infection, haematological malignancy, or autoinflammatory disorders. The spectrum of disease in PAN ranges from single-organ involvement to multi-organ failure. As a result, PAN has a broad spectrum of possible clinical manifestations. Constitutional symptoms, such as fever and myalgia, are the most common. However, neurological, renal, dermatological, gastrointestinal, cardiovascular, and pulmonary manifestations are also reported. While cardiac involvement is well-described and is a common cause of mortality, it is exceedingly uncommon as the initial presentation. Below, we describe a case of a female in her 60s who presented with pericarditis as the first manifestation of PAN.

## Case presentation

A woman in her 60s was transferred to the coronary care unit (CCU) from the local accident and emergency department with a three-day history of pleuritic central chest pain, which was positional in nature with associated intermittent palpitations. She denies previous similar episodes and reports no syncope, pre-syncope, recent illness, or shortness of breath. The patient has a past medical history of Raynaud’s phenomenon, gastroesophageal reflux, and mild sleep apnoea for which she uses nocturnal continuous positive airway pressure. At the time, the patient was not taking any regular medications and reported no known allergies. The patient is a retired, independent, non-smoker with minimal alcohol consumption. The patient's mother passed away from aortic dissection, and one of the patient's children has a diagnosis of ulcerative colitis.

**Figure 1:**
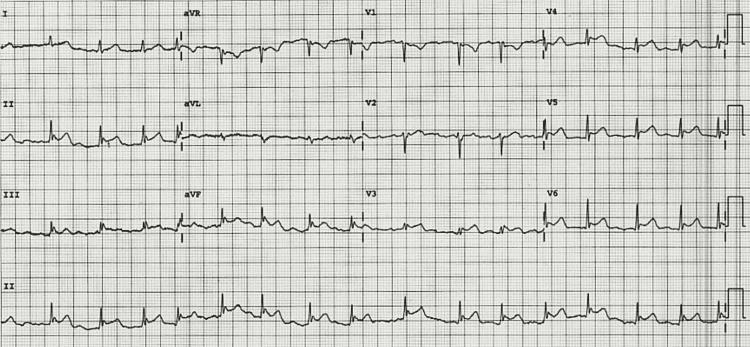
ECG - Pericarditis and atrial fibrillation ECG showing widespread ST elevations with concave morphology and irregular rhythm with no P waves consistent with Atrial Fibrillation and pericarditis.

Her ECG on presentation in Figure 1 showed an irregularly irregular rhythm with a lack of P waves, widespread ST-segment elevation with concave morphology, no reciprocal changes, subtle PR elevation in lead aVR, and PR depression in leads aVF and 2. These ECG features in the clinical context are highly suggestive of pericarditis with concurrent atrial fibrillation.

**Figure 2:**
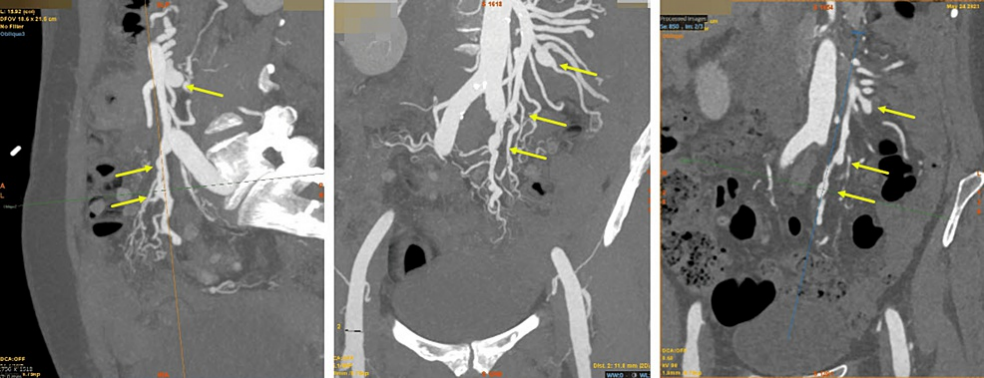
CT - Mesenteric aneurysms 10 mm fusiform aneurysm of the jejunal branch of the superior mesenteric artery, and 9 mm distal superior mesenteric artery aneurysm with further small areas of focal vascular ectasia of the ileal branches (arrows)

During the evening of admission, the patient complained of chest pain radiating to the back during an episode of hypotension (BP 80/45). Hence, a CT aorta was performed, which excluded aortic dissection. Instead, the CT aorta showed aneurysms throughout the mesenteric vascular system (Figure 2). Aneurysmal coronary arteries, diffusely aneurysmal right hepatic artery, axillary aneurysms, bi-apical pulmonary fibrosis, and shallow pericardial effusion were also noted.

The patient was treated for pericarditis with colchicine and ibuprofen, anticoagulated for atrial fibrillation with rivaroxaban (initiated one-week post-discharge), and rate-controlled with bisoprolol. Based on the CT findings and presentation, an underlying aetiology of vasculitis was suspected. As a result, the patient was reviewed by the rheumatology service, who advised on vasculitis and infectious screenings. The results of which are seen in Table 1.

**Table 1:** Blood tests Key: DC = discharge; OP = outpatient; F/U = Follow-up

Initial Admission Blood tests										
Routine	1st Admission	1st DC		OP F/U		2nd Admission	2nd DC		Ref Range	
WBC	10.70	6.90		7.70		10	10.4		4.0-11.0	
Haemoglobin	110.00	124.00		120.00		117	106		115-150	
CRP	166.00	118.00		20.00		104	101		0-10	
eGFR	>90	>90		>90		>90	>90			
Urea	3.5	2.8		4.4		3.5	4.4		2.5-7.8	
Sodium	129.00	135.00		133.00		136	136		133-146	
Potassium	4.50	4.60		4.30		4.6	4		3.5-5.3	
Other										
TSH	1.10									
Troponin (2nd)	23 (27)									
D dimer	0.70								0.00-0.50	
INR	1.20									
Creatinine Kinase	42.00								25-200	
Vasculitis screen										
ANA	Negative									
ENA (Ro, La, Sm, U1RNP, RNP70, CENP, Jo-1, Scl-70)	Negative									
Rheumatoid factor	9.00								0-12.5	
MPO-ANCA	<1.0								0-6	
PR3-ANCA	<0.6								0-5	
C3	1.30								0.75-1.65	
C4	0.27								0.14-0.54	
Infectious screen										
HIV 1 & 2 combined ab/ag test	Not detected by EIA									
Hepatitis B	Not detected by EIA									
Hepatitis C	Not detected by EIA									
QuantiFERON TB Gold Plus	Negative									
QuantiFERON Antigen 1	0.00 IU/mL									
QuantiFERON Antigen 2	0.00 IU/mL									
QuantiFERON Mitogen	1.00 IU/mL									
Glandular fever screen	Negative									

Good clinical and biochemical improvement was seen in response to this treatment (Table 1). The patient was discharged and followed up in the rheumatology clinic several days after discharge.

Following this, 15 days after the initial discharge, the patient represented to the hospital with pre-syncope and shortness of breath. Her ECG at the time showed electrical alternans consistent with significant pericardial effusion.

**Figure 3:**
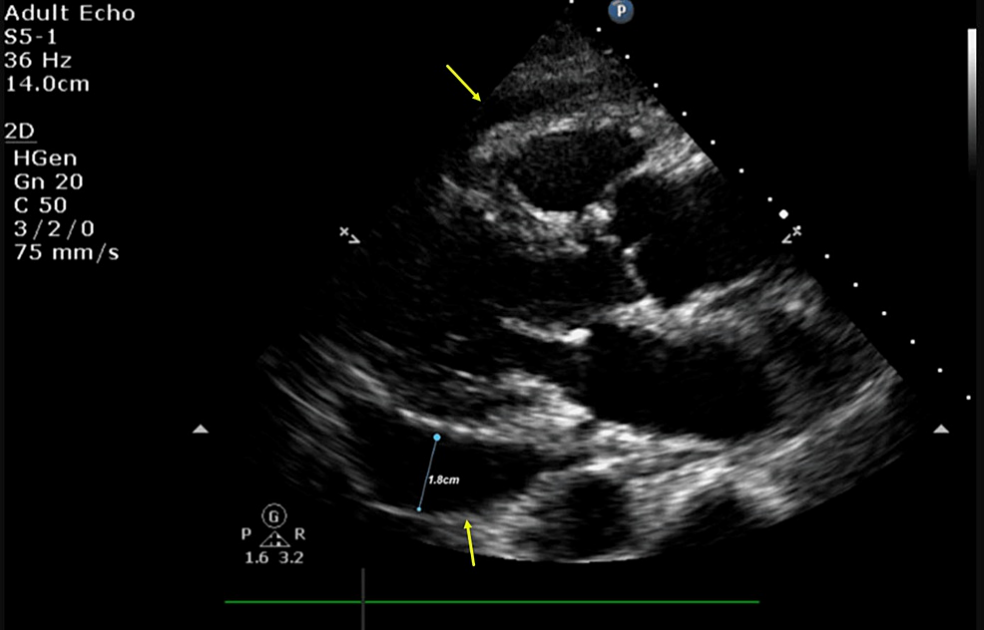
Echocardiogram with large pericardial effusion Echocardiogram image showing global pericardial effusion around the left and right ventricles in the parasternal long-axis view. Effusion measuring 1.8 cm around the left ventricle (arrows).

Her echocardiogram confirmed a large pericardial effusion with features of incipient cardiac tamponade, as seen in Figure 3. Her pericardial effusion progressed, and right ventricular collapse developed, causing haemodynamic compromise. As a result, a pericardial drain was placed, and 450 ml of slight pink straw-coloured pericardial fluid was drained. The drain was removed two days later with a resolution of the pericardial effusion.

After removing the pericardial drain, the patient had an uneventful stay free of complications. A diagnosis of polyarteritis nodosa was made on the basis of the clinical features and angiographic findings. As a result, the patient was started on prophylactic Co-trimoxazole, a reducing prednisolone dose and bone protection medication. The patient was discharged with the plan to start cyclophosphamide as an outpatient with rheumatology follow-up. The patient has since reported intermittent episodes of asymptomatic low blood pressure, palpitations, and chest discomfort. Hence, she has remained under cardiology and rheumatology follow-up as an outpatient.

## Discussion

Acute pericarditis is a relatively common presentation that accounts for 5% of non-ischaemic chest pain in emergency departments ​[3,4]​. It is generally considered self-limiting and not life-threatening when myocardial infarction, dissection, and cardiac tamponade have been excluded ​[4]​. Most cases are considered viral in origin, and the exact aetiology is not identified in most cases ​[4]​. Such cases are labelled as idiopathic pericarditis. Most idiopathic pericarditis responds to conventional treatment with non-steroidal anti-inflammatory medication and colchicine, with little to no reoccurrence ​[5]​. This case highlights the importance of recognising the autoimmune causes of pericarditis, such as PAN. Such cases of pericarditis may not have sustained response to conventional treatment, which may predispose them to relapse and more severe complications such as cardiac tamponade, as seen in this case. Furthermore, if left untreated, there is approximately only a 13% five-year survival rate in PAN ​[6,7]​. With prompt recognition and treatment with modern immunotherapy (e.g., steroids and cyclophosphamide), PAN's five- and 10-year survival rates improve to around 83% and 74%, respectively ​[8]​. 

The variety of clinical presentations and symptoms that can be seen in PAN is shown in Table 2. Cardiac manifestations of PAN have been documented since the first case report from Kussmaul and Maier (1866), who described nodular coronary inflammation ​[9]​. In histological studies at autopsy in 1962, pericardial involvement was reported to be as high as 19% ​[10]​. However, over 90% of these cases were pericarditis secondary to uraemia related to renal failure ​[10]​. A histological retrospective review of 66 cases of PAN from 1926 to 1958 found 22 cases of PAN to have evidence of pericarditis​ [11]​. Of these 22, nine (13%) cases were considered to have non-uremic pericarditis or pericarditis directly caused by polyarteritis nodosa, also known as pericarditis of PAN​ [11]​. The Chapel Hill Consensus Conference (CHCC) formally distinguished microscopic polyangiitis and PAN in 1994 ​[12]​. Hence, reports on the incidence, prevalence, and manifestations of PAN in the above studies, which predate 1994, must be interpreted cautiously, as many cases of microscopic polyangiitis were likely included. Modern studies suggest that pericarditis, as a clinical manifestation of PAN, is reported in around 5% of cases ​[8]. Although this study did not mention pericarditis related to uraemia, 66% of the cohort had renal involvement. Hence, the number of patients with pericarditis secondary to uraemia or primary pericarditis of PAN is unknown. Generally, pericarditis of PAN, such as in our case, is uncommon. To the author's knowledge, pericarditis as the first presentation of PAN has yet to be reported.

**Table 2:** Clinical manifestations Adapted from Ref. (8)

Clinical Manifestations of Polyarteritis Nodosa	
Manifestation	Frequency (%)
Constitutional *(Weight loss, myalgia, fever)*	93
Neurological *(Neuropathy, Stroke, Delirium)*	79
Urological *(Orchitis, haematuria)*	50
Dermatological *(nodules, purpura, livedo)*	50
Renal injury	51
Joint pain	49
Gastrointestinal *(abdominal pain, bleeding, perforation, cholecystitis, appendicitis, pancreatitis)*	38
Hypertension	34
Cardiovascular *(cardiomyopathy, pericarditis, digital ischaemia)*	22
Pulmonary	12
Ophthalmologic	9

There is no consensus on the pathogenetic mechanism of PAN. It is thought that the wide variation in clinical manifestations seen in Table 2 likely represents multiple separate pathogenic mechanisms from a spectrum of underlying diseases, resulting in the same constellation of clinical phenotypic features​ [13]​. Over time many underlying disease states have been associated with PAN, further supporting this theory. Table 3 summarises the possible disease associations. The most well-known form of secondary PAN is caused by hepatitis B infection​ [14]​. Hepatitis C and hairy cell leukaemia have also been reported to play a role in the pathogenesis of some cases​ [15,16]​. PAN-like vasculitis has also been reported in monogenic auto-inflammatory conditions such as familial Mediterranean fever, adenosine deaminase-2 deficiency, and VEXAS syndrome​ [14,17]​. 

**Table 3:** Conditions associated with PAN Adapted from Ref.​ (14)​

Pathologies Associated with Polyarteritis Nodosa or Polyarteritis Nodosa-Type Vasculitis	
		Infection	
			Hepatitis B
	Hepatitis C
	Parvovirus B19
	HIV
	EBV
Haematological disorders	
	Hairy cell leukemia
	Myelodysplastic syndromes
Monogenic causes	
	Familial Mediterranean fever
	Adenosine deaminase-2 deficiency
Neoplasia	

The incidence of PAN has decreased with the identification of secondary causes and the delineation of idiopathic PAN. Contemporary evidence suggests the incidence of PAN is 0-1.6 cases per million per year, and the prevalence is up to 33 cases per million in European populations​ [1]​. Although PAN can affect patients of any gender, age, or ethnicity, its peak incidence is in the fifth and sixth decades of life, as seen in our patient [1,2]​.

With no common consensus or validated diagnostic criteria, the diagnosis of PAN is largely based on symptom recognition, clinical suspicion, imaging, biopsy, and the exclusion of secondary causes​ [14]​. The American College of Rheumatology criteria for the classification of PAN, seen in Table 4, can aid the diagnosis and clinical decision for further investigation [18]​.

**Table 4:** ACR PAN classification criteria Adapted from ​Ref. (18)​ PAN, polyarteritis nodosa ACR, American College of Rheumatology

American College of Rheumatology PAN Classification Criteria
Weight loss > 4 kg
Livedo reticularis
Testicular pain or sensitivity
Diffuse myalgia, muscle weakness, or leg tenderness
Mono or polyneuropathy
Diastolic pressure > 90mm Hg
Renal failure (BUN > 14.3 mmol / L or serum creatinine > 132 umol/L)
Presence of HBV antigen or HBV antibodies in serum
Angiographic abnormalities (aneurysm and/or occlusion of visceral arteries)
Biopsy of a small or medium-sized artery showing neutrophils in the arterial wall

Ideally, the diagnosis should be confirmed histologically with a biopsy of an affected organ​ [14]​. However, muscle and nerve biopsies diagnose 75% of cases, renal biopsies are not advised, and cutaneous biopsies show vasculitis in roughly 50% of patients ​[14]​. When a biopsy is not possible such as in our case, the diagnosis can be made through angiography​ [14,19]​. Angiographic evidence of mesenteric, hepatic, and renal focal segmental aneurysms is seen in up to 90% of cases of PAN ​[14]. Current guidelines recommend CT or MR angiography as noninvasive diagnostic techniques for diagnosis, baseline assessment of disease activity, and determining disease severity [19]​.

## Conclusions

Pericarditis is often considered a relatively benign condition where no specific cause is often found. However, this case shows that not all cases of pericarditis are self-limiting, underscoring the importance of identifying an aetiology and ruling out systemic diseases, such as vasculitis. Pericarditis caused by vasculitis, specifically PAN, can be refractory to conventional pericarditis treatment and potentially life-threatening. Modern treatment significantly reduces the mortality associated with PAN, highlighting the importance of identifying the aetiology. Despite once a problematic diagnosis to confirm, current guidelines suggest that CT angiography can help confirm the diagnosis, as illustrated in this case. Vasculitis such as PAN should be considered in cases of pericarditis with evidence of multisystem involvement.
